# Evaluation of genetic risk score models in the presence of interaction and linkage disequilibrium

**DOI:** 10.3389/fgene.2013.00138

**Published:** 2013-07-23

**Authors:** Ronglin Che, Alison A. Motsinger-Reif

**Affiliations:** Department of Statistics, Bioinformatics Research Center, North Carolina State UniversityRaleigh, NC, USA

**Keywords:** explained variance, genetic risk score (GRS), interaction, linkage disequilibrium (LD), predictive modeling

## Abstract

In the area of genetic epidemiology, genetic risk predictive modeling is becoming an important area of translational success. As an increasing number of genetic variants are successfully discovered, the use of multiple genetic variants in constructing a genetic risk score (GRS) for modeling has been widely applied using a variety of approaches. Previously, we compared the performance of a simple, additive GRS with weighted GRS approaches, but our initial simulation experiment assumed very simple models without many of the complications found in real genetic studies. In particular, interactions between variants and linkage disequilibrium (LD) (indirect mapping) remain important and challenging problems for GRS modeling. In the present study, we applied two simulation strategies to mimic various types of epistasis to evaluate their impact on the performance of the GRS models. We simulated a range of models demonstrating statistical interaction and linkage disequilibrium. Three genetic risk models were compared in terms of power, type I error, C-statistic and AIC, including a simple count GRS (SC-GRS), an odds ratio weighted GRS (OR-GRS) and an explained variance weighted GRS (EV-GRS). Simulation factors of interest included allele frequencies, effect sizes, strengths of interaction, degrees of LD and heritability. We extensively examined the extent to how these interactions could influence the performance of genetic risk models. Our results show that the weighted methods outperform simple count method in general even if interaction or LD is present, with well controlled type I error.

## Introduction

In recent years, Genome-Wide Association Studies (GWAS) and candidate gene studies have identified a large number of genetic variants with varying effect size that are associated with complex diseases (McCarthy et al., 2008) and drug response/pharmacogenomic traits (Ritchie, 2012). This identification of causal and/or associated variants provides new opportunities to develop more personalized approaches to disease prediction and prevention. One popular approach for incorporating identified genetic variants is by constructing a genetic risk score (GRS) for modeling using a variety of approaches, such as an additive simple count and weighted GRS (Carayol et al., 2010; Paynter et al., 2010). The applicability of these cumulative risk scores as predictive models for disease has been proposed and brought anecdotal successes in the real genetic studies (Hess et al., 2006; Meigs et al., 2008; Klein et al., 2009; Manolio, 2010). While several approaches have been implemented successfully and become widely applied in real data analyses, these risk score models have not been thoroughly evaluated, particularly in complex scenarios.

Previously, we compared the performance of a simple, additive GRS with weighted GRS approaches in a wide range of simulation scenarios. Our initial findings show that a weighted method involving both the odds ratio and allele frequency of variants robustly outperforms other GRS models in general (Che and Motsinger-Reif, 2012). However, we recognized that our simulation experiment assumed very simple models without many of the complications found in real genetic studies. In particular, interactions between variants in predicting disease risk and linkage disequilibrium (LD) (indirect correlation between markers) remain important and challenging problems (Winham et al., 2012). When moving from variant discovery to validation and prediction, such complex architecture may bring more challenges. Therefore, a more comprehensive exploration of the consequence of complex etiology and multi-locus correlation on risk modeling is becoming a priority. Although the primary goal of GRS is beyond the initial detection of risk alleles, and typically only involves variants with previously established associations, ignoring interactions may largely limit the success of risk prediction model for complex disease and pharmacogenomic studies.

Epistasis or gene-gene interaction has become a hot topic in complex disease genetics recently. In the previous literature, discussion of epistasis has been considerably confused by differing definitions as well as by applying the same terminology to quite different concepts. In essence, epistasis refers to departure from “independence” of the effects of multiple loci in the way that they combine to cause disease (Cordell, 2002). From a statistical point of view, epistasis represents departure from additivity in a mathematical model that describes the relationship between multiple variants and disease outcome in the population (Cordell, 2002, 2009; Pattin et al., 2009). In contrast, we also simulated models that exhibit correlation between loci—representing linkage disequilibrium (LD) (Lunetta, 2008). Linkage disequilibrium refers to the non-random association of alleles at two or more loci in a population (Shifman et al., 2003; Lunetta, 2008).

In the current study, we explicitly compared the performance of three genetic risk score approaches to detect the true risk model in the presence of interaction and LD, including a simple count (SC-), an odds ratio weighted (OR-) and an explained variance weighted (EV-) genetic risk score method. We employed two simulation strategies to represent (1) epistasis (gene-gene interaction effects on the phenotype) and (2) linkage disequilibrium. The effect sizes and allele frequencies were considered as important factors to evaluate the role of weights in the GRS construction. We also addressed the relationship among effect size, allele frequency, interaction effect, heritability and the predictive power for the GRS models. The main goal of this study was to explore the relationship between the degree of interaction and the performance of genetic risk scores in model prediction in a wide range of scenarios, which may in turn help guide the application of GRS in risk prediction.

## Methods

### Genetic risk score models

In the present study, GRS models were evaluated for single nucleotide polymorphism (SNP) data only. However, it could be easily generalized to other genetic variants or risk factors. It should be noted that we choose SNPs in the GRS construction based on either the previous knowledge of the disease predisposition or the data at hand. Several promising approaches, such as random forests (Winham et al., 2012) and statistical epistasis networks (Hu et al., 2011), were developed to characterize genetic interactions. An evaluation of methods for the identification and selection of risk variants with interaction are beyond the primary goal of the GRS models as implemented here. In the current study, we assume the associated SNPs have been discovered prior to GRS modeling.

Three representative GRS methods were compared in this study, including SC-, OR- and EV-GRS. As implemented in a previous study, a very simple main effect and additive genetic model was assumed in the risk modeling (Che and Motsinger-Reif, 2012). Let *D* denote the binary phenotype value, where *D* = 1 represent case and *D* = 0 represent control. **G** is a vector of genotype combinations across all genetic loci. *G*_*i*_ is the number of the risk alleles for the i-th SNP. α is an intercept and β measures the overall effect of the genetic risk score. All parameters were estimated under a logistic regression model without any interaction term (Che and Motsinger-Reif, 2012).

#### Simple count GRS (SC-GRS)

(1) $$\text{logit}P(D=1|G)=\alpha +\beta (SC\_GRS)=\alpha +\beta \sum_{i=1}^{I}G_{i}$$

(2) $$SC\_GRS=\sum_{i=1}^{I}G_{i}$$

This simple count score model sums up all risk alleles over all loci as a summary score. No prior knowledge is needed. It is relatively simple and thus is in wide application in the current literature (Paynter et al., 2010).

#### Odds ratio weighted GRS (OR-GRS)

(3) $$\text{logit}P(D=1|G)=\alpha +\beta (OR\_GRS)=\alpha +\beta \sum_{i=1}^{I}w_{OR\_i}G_{i}$$

(4) $$w_{OR\_i}=\log(OR_{i})$$

(5) $$OR\_GRS=\sum_{i=1}^{I}w_{OR\_i}G_{i}$$

(6) $$\text{rescaled}:OR\_GRS=I\left(\sum_{i=1}^{I}w_{OR\_i}G_{i}\right)\text{​​}/\text{​​}\left(\sum_{i=1}^{I}w_{OR\_i}\right)$$

This weighted summary score takes into account the fact that effect sizes among SNPs vary. In general, log per-allele odds ratio from meta-analysis or from other independent data is considered as a reasonable weight to apply to each SNP (Talmud et al., 2010). A rescaled score is utilized since it is more directly comparable to the unweighted score.

#### Explained variance weighted GRS (EV-GRS)

(7) $$\text{logit}P(D=1|G)=\alpha +\beta (EV\_GRS)=\alpha +\beta \sum_{i=1}^{I}w_{EV\_i}G_{i}$$

(8) $$w_{EV\_i}=\log(OR_{i})\sqrt{2MAF_{i}(1-MAF_{i})}$$

(9) $$EV\_GRS=\sum_{i=1}^{I}w_{EV\_i}G_{i}$$

(10) $$\text{rescaled}:EV\_GRS=I\left(\sum_{i=1}^{I}w_{EV\_i}G_{i}\right)\text{​​}/\text{​​}\left(\sum_{i=1}^{I}w_{EV\_i}\right)$$

An alternative weight incorporates MAF as well as OR. The development of this score was motivated by the assumption that both MAF and OR are important factors to explain the genetic variance and heritability (Park et al., 2010). It has been developed to construct a weight in the risk modeling. The odds ratio estimates could be obtained identically as discussed above for the OR-GRS, and the MAF could be generated from NCBI and HapMap databases, or from the data at hand.

### Simulation design

#### Simulation design 1: interaction

In the current study, we simulated epistasis as multiple loci, non-additive disease risk models. In these simulations, the loci involved in the interaction model are independent (not correlated), but together interact to predict disease. This “statistical interaction” was simulated assuming that the multiple loci genotypes jointly contribute to an underlying (unobserved) continuous trait by varying their interaction term under a linear regression model. The disease occurs if this continuous trait exceeds a certain threshold.

Firstly, genotype *G*_*i*_ at SNP *i* was generated independently under Hardy-Weinberg Equilibrium (HWE). The genotype value was coded as 0, 1, and 2, representing the number of risk alleles. Minor allele frequencies (MAF) of the SNPs were set to 0.4 and 0.05, which resemble common and relatively rare variants. Continuous phenotypes *Y* were then generated conditional on genotypes, according to

(11) $$\begin{array}{l} Y=\beta_{0}+\beta_{1}G_{1}+\beta_{2}G_{2}+\beta_{3}G_{3}+\beta_{4}G_{4}\\ \;+\;\beta_{34}G_{3}G_{4}+e,e\sim N(0,\sigma^{2}) \end{array}$$

where β_0_ = 20 and σ^2^ = 10. Binary affection status *D* was assigned to 1 (case) if phenotype *Y* > *median(Y)* and *D* was 0 (control) otherwise, where the threshold *median(Y)* was only chosen in order to achieve prevalence P(D) = 0.5 and balanced case and control data. Under this linear model, β = (β_0_, β_1_, β_2_, β_3_, β_4_, β_34_) was ranged to reflect different effect sizes (ES) among SNPs. SNPs 1 and 2 have main effects only, while SNPs 3 and 4 have interactions where β_34_ reflects the strength of interactions (Winham et al., 2012). In this simulation, main effect means SNPs contribute to the disease risk in an independent way only, while interaction effect defines SNPs are independent but attribute to the risk both marginally and dependently.

We considered 2 scenarios in simulation one, with 4 and 2 disease-causing SNPs respectively, with a total of 4 SNPs simulated. In scenario 1, our primary interest was to compare the performance of genetic risk models including only true “deleterious” SNPs. Four SNPs were causative with both main and interaction effects. In scenario 2, only SNPs 3 and 4 were causative with interaction effects. The priority was to investigate risk models when “noise” SNPs were present. In order to examine the effect of weight and interaction, important simulation factors involved MAF, effect size (ES) and interaction. All common variants, all rare variants, common plus rare variants were simulated with both same and different effect sizes among these SNPs, and three settings of interactions (negligible, moderate and strong) were considered, which led to 18 combinations in total. For each combination, 100 replicates were generated with 250 cases and 250 controls. In preliminary studies we simulated datasets with sample sizes of 1000. The results showed similar patterns and thus the additional details were not included.

Heritability in the broad sense is defined as the proportion of the phenotypic variance that could be attributed to variance of genotypic values, and in the narrow sense it is due to the additive genetic effect (Visscher et al., 2008). The specific heritability component mainly depends on MAF, ES and degree of interaction. Culverhouse et al. and Winham et al. have described the calculation of heritability in the presence of main effects and epistasis for binary traits as simulated here (Culverhouse et al., 2002; Winham et al., 2012). For a single locus A with genotypes *a* = 0, 1, 2, the heritability can be expressed as:

(12) $$H_{A}^{2}=\frac{\sum_{a=0}^{2}P(G_{a}){\left\{P(D|G_{a})-P(D)\right\}}^{2}}{P(D)\{1-P(D)\}},$$

where *P*(*G*_*a*_) is the genotype frequency of locus A, *P*(*D*) is the disease prevalence and *P*(*D*|*G*_*a*_) is the penetrance. Penetrance was defined as the probability of disease conditional on a particular genotype combination at the disease risk locus/loci. Similarly, the total heritability due to the two loci A and B, with genotypes *a*, *b* = 0, 1, 2 respectively, is:

(13) $$H_{AB}^{2}=\frac{\sum_{a=0}^{2}\sum_{b=0}^{2}P(G_{ab}){\left\{P(D|G_{ab})-P(D)\right\}}^{2}}{P(D)\{1-P(D)\}},$$

where *P*(*G*_*ab*_) is the frequency of genotype combinations of *a* and *b*, and *P*(*D*|*G*_*ab*_) is the corresponding penetrance. The heritability due to the marginal effect of locus A is defined as:

(14) $$H_{M,\;A}^{2}\text{​}=\text{​}\frac{\sum_{a=0}^{2}\left\{\sum_{b=0}^{2}P(G_{ab})\right\}{\left\{\sum_{b=0}^{2}P(D|G_{ab})P(G_{ab})\text{​}-\text{​}P(D)\right\}}^{2}}{P(D)\{1\text{​}-\text{​}P(D)\}}.$$

The heritability due to the interaction effect could therefore be interpreted as the proportion of that is not attributable to the marginal effects by either locus, that is:

(15) $$H_{I,\;AB}^{2}=H_{AB}^{2}-H_{M,\;A}^{2}-H_{M,\;B}^{2}.$$

This heritability calculation method could be generalized to a scenario with more causal SNPs. The property of our simulated models could be reflected by varying heritability component due to the main effects (*H*^2^_*G1G*2_ = *H*^2^_*M*, *G*1_ + *H*^2^_*M*, *G*2_) and interaction effects (*H*^2^_*G*3*G*4_ = *H*^2^_*M*, *G*3_ + *H*^2^_*M*, *G*4_ + *H*^2^_*I*, *G*3*G*4_). Low heritability (*H*^2^_*Total*_ = *H*^2^_*G*1*G*2_ + *H*^2^_*G*3*G*4_ < 5%) was chosen to reflect detectable effect sizes in the realistic genetic association studies and to ensure the reasonable power to discriminate the difference of performance (Winham et al., 2012).

Supplemental Table S1 summarized the simulated model specifications in terms of MAF, ES, interaction and heritability.

#### Simulation design 2: linkage disequilibrium

In simulation two, SNP linkage disequilibrium (correlation) was investigated. We assumed two loci were correlated regardless of disease outcome. To mimic linkage disequilibrium, simulations were performed by designing the genotype combination frequencies of two or more loci, and in turn contributing to disease in an additive but relatively independent fashion under a probabilistic model. We simulated a true disease risk model involving two independent “deleterious” SNPs (SNPs 1 and 2). Two scenarios were considered with SNP 3 was dependent (strong LD model) and independent (weak LD model) of SNP 2 respectively. SNPs 1 and 2 were generated as multinomial under HWE, and then SNP 3 was generated according to Supplemental Table S4. In Scenario 1, for each level of SNP 2 values, the genotype frequencies of SNP 3 varied. We defined that SNPs 2 and 3 were in strong LD (correlated). In Scenario 2, the genotype frequencies of SNP 3 were fixed and irrelevant to the genotype of SNP 2. In this case, we defined SNPs 2 and 3 were in weak/no LD (non-correlated). GRS approaches were compared when only “deleterious” SNPs were included (true model), or when all three SNPs were included (Scenario 1 strong LD: true + dependence model; Scenario 2 weak LD: true + independence model). In the case of weak/no LD, including the third SNP could also be thought of as including a false positive locus.

Rather than the linear model in simulation one, a direct probabilistic model was used here to generate the disease status. To simplify, an additive genetic mode was determined. Let the alleles from SNP 1 be denoted “A” and “a”, and let those from SNP 2 be denoted “B” and “b”. Then, SNP 1 has three genotypes as “AA”, “Aa” and “aa”, whereas SNP 2 has three genotypes as “BB”, “Bb” and “bb” correspondingly, thus leading to nine genotypes combinations. Table 1 demonstrated the penetrance pattern for two-locus main effect model for nine genotypes combinations, where *k* was the baseline penetrance and θ was the specified relative risk (RR) of having a disease between different genotypes for each SNP.

**Table 1:** **Penetrance pattern under additive genetic mode for two-locus main effect model**.

**Mode**	**Genotype**	BB	Bb	bb
Additive	AA	*k*	$\frac{(\theta_{b}+1)k}{2}$	θ_*b*_ *k*
	Aa	$\frac{(\theta_{a}+1)k}{2}$	$\frac{(\theta_{a}+\theta_{b})k}{2}$	$\frac{(\theta_{a}+2\theta_{b}-1)k}{2}$
	aa	θ_*a*_ *k*	$\frac{(2\theta_{a}+\theta_{b}-1)k}{2}$	(θ_*a*_ + θ_*b*_ − 1)*k*

As presented in Supplemental Table S5, MAFs ranged from 0.4 to 0.05 to represent common and relatively rare variants. Four scenarios were included, one where both SNPs 1 and 2 were common or rare, one where SNP 1 was common and SNP 2 was rare, and vice versa. Relative risks (RRs) considered in our model were either 1.25 or 1.75 to simulate different effect sizes between SNPs 1 and 2, and 1.5 to simulate models where both SNPs had the same effect sizes. Baseline penetrance *k* was fixed at 0.1. Balanced (equal allocation) case-control data was simulated with a total sample size of 400. 100 replicates were simulated as training data to calculate external weights for OR- and EV-GRS approaches, and then another 100 replicates were generated as test data to evaluate the performance of GRS methods.

### Data analysis

The main focus of the prediction modeling was to determine the true risk model correctly. In general, the training data sets were used to derive weights for the weighted GRS methods, assuming all causative SNPs are known. For each method, the GRS were calculated for each subject in the test data. A logistic regression model was applied to fit the test data using each genetic risk modeling approach respectively, with a summary GRS as the only predictor. We used power, type I error, C-statistic and Akaike information criterion (AIC) to evaluate the performance of each of the GRS. Power or type I error rate was calculated as the number of times the model is statistically significant at *P*-value <0.05 across the number of simulated replicates (Che et al., 2012). A likelihood ratio test was used as the global measure of model fit. In the simulation study, if the binary case/control status was assigned based on the specific genetic values, the detection ability could be reflected by power. In contrast, if the case/control status was irrelevant to genetic variants, the type I error under the null simulation then could be interpreted as false positive rate. The best risk score approach was expected to detect the model if it is true, whereas to control the probability to incorrectly accepting the false model. The C-statistic measures the discriminatory capability of each model to distinguish case from control. AIC is a measure of the goodness of fit of the model, which describes the tradeoff between accuracy and complexity of the model (Che et al., 2012). In general, a model is preferred if it has larger power and C-statistic and has a smaller AIC, with a reasonable type I error.

Under the null model (involving only “noise” SNP), the *P*-value for the likelihood ratio test was recorded for each replicate, and then the number of times across the 100 replicates that the *P*-value was less than 0.05 was calculated as the type I error. Under the true disease risk model (involving any causative SNP), the C-statistic, AIC and *P*-value for the likelihood ratio test were recorded. The power was calculated as the proportion of times a true model was correctly identified (*P*-value < 0.05) across 100 replicates. For each model, the C-statistic and AIC were averaged across all replicates. All results were statistically evaluated for differences under a generalized linear model, and Tukey's method was used to adjust for pair-wise contrasts between methods, which has been described previously (Winham et al., 2010; Che and Motsinger-Reif, 2012).

In simulation one, data generation was performed on R platform (www.r-project.org). For all the remaining data simulations and analyses were applied using SAS 9.2 (www.sas.com).

## Results

### Simulation result 1: interaction

**Figure 1:**
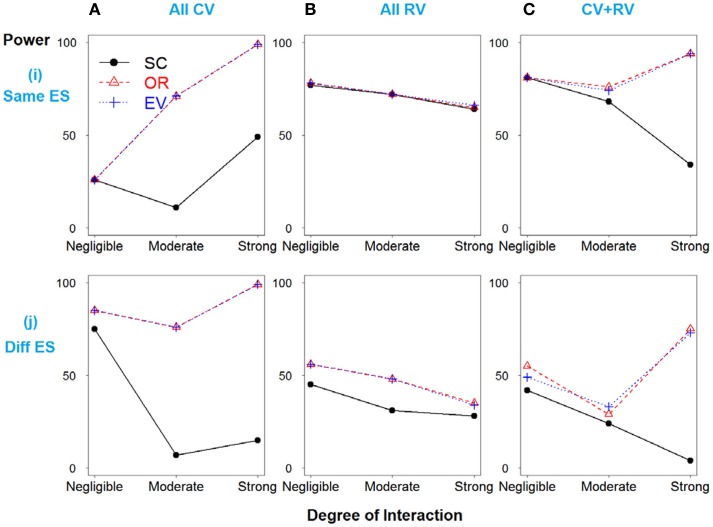
**Power comparison for the interaction model with four deleterious SNPs**. Panels by column: **(A)** All SNPs are common variants. **(B)** All SNPs are rare variants. **(C)** SNPs 1 and 3 are common variants, and SNPs 2 and 4 are rare variants. Panels by row: **(i)** SNPs have same effect size. **(j)** SNPs have different effect size.

In Scenario 1, with four deleterious SNPs, both main and interaction effects exist. Figure 1i shows the power results when all the SNPs have similar effect sizes. If there is no interaction between SNPs 3 and 4, the weighted and unweighted methods are equivalent. When all SNPs are common variants (Figure 1A) or both common and rare variants (Figure 1C), as the degree of interaction increases, the weighted methods outperform the simple count GRS. However, when all SNPs are rare variants, the power of three methods is identical.

In Figure 1j, when the effect sizes among SNPs vary, it is clear that the weighted methods are consistently preferable to the unweighted one. There is no significant difference between the two weighted methods, OR- and EV-GRS.

**Figure 2:**
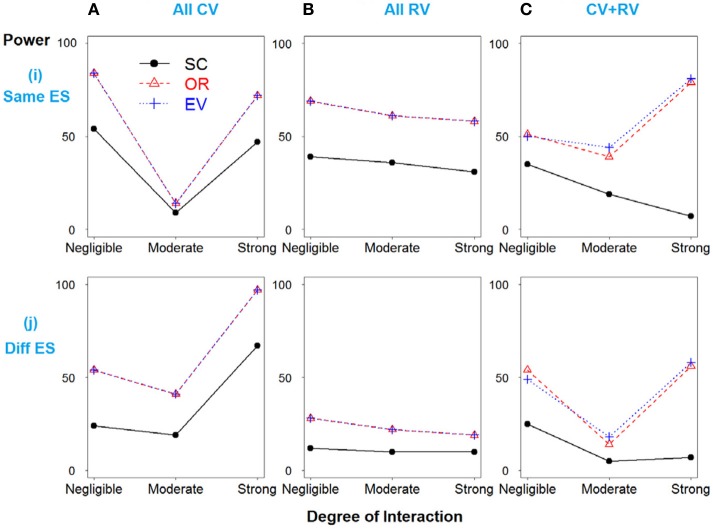
**Power comparison for the interaction model with two deleterious SNPs**. Panels by column: **(A)** All SNPs are common variants. **(B)** All SNPs are rare variants. **(C)** SNPs 1 and 3 are common variants, and SNPs 2 and 4 are rare variants. Panels by row: **(i)** SNPs have same effect size. **(j)** SNPs have different effect size.

In Scenario 2, the total heritability is due to SNPs 3 and 4 only, and then SNPs 1 and 2 were simulated as “noise” SNPs. As expected, the weighted methods show improved power over the simple count method across all models (Figure 2).

### Simulation result 2: linkage disequilibrium

**Figure 3:**
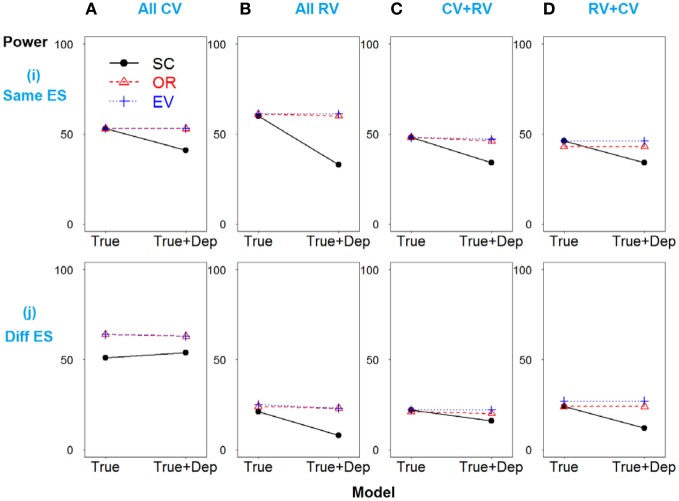
**Power comparison for the strong linkage disequilibrium model when SNPs 2 and 3 are dependent**. Panels by column: **(A)** SNPs 1 and 2 are common variants. **(B)** SNPs 1 and 2 are rare variants. **(C)** SNP 1 is common variant, and SNP 2 is rare variant. **(D)** SNP 1 is rare variant, and SNP 2 is common variant. Panels by row: **(i)** SNPs have same effect size. **(j)** SNPs have different effect size. “Dep” means SNPs 2 and 3 are dependent.

Scenario 1 shows the strong linkage disequilibrium model when SNPs 2 and 3 are correlated. As demonstrated in Figure 3i, all three methods have similar power if only deleterious SNPs with similar effect sizes are included in the risk model (true model). However, if the dependent but non-causative SNP 3 is added (so a model including true loci plus marker(s) that are correlated with the true loci), the power of SC-GRS declines rapidly.

For the scenarios where there are different effect sizes of the causative SNPs (Figure 3j), the results of the power comparison are very similar, except the weighted methods are also preferable when the causal variants are common (Figure 3Aj).

**Figure 4:**
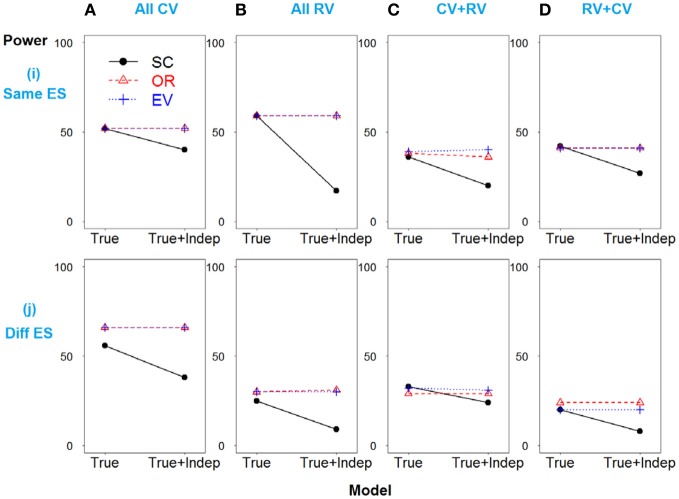
**Power comparison for the weak linkage disequilibrium model when SNPs 2 and 3 are independent**. Panels by column: **(A)** SNPs 1 and 2 are common variants. **(B)** SNPs 1 and 2 are rare variants. **(C)** SNP 1 is common variant, and SNP 2 is rare variant. **(D)** SNP 1 is rare variant, and SNP 2 is common variant. Panels by row: **(i)** SNPs have same effect size. **(j)** SNPs have different effect size. “Indep” means SNPs 2 and 3 are independent.

A similar pattern is observed when SNP 3 is independent and non-causative in the weak linkage disequilibrium model, as shown in Figure 4.

In summary, the weighted approaches outperform the SC-GRS across all scenarios in terms of power and C-statistic (*P*-value <0.05), as shown in Supplemental Tables S6, S7. EV- is slightly better than OR-GRS, with no significant difference. In respect to AIC, there is no significant difference.

For the null simulations, the type I error rates across all models are well reasonably controlled (data was shown in Supplemental Tables S3, S5). There are no statistically significant differences in terms of type I error among these methods and the cut-off value 0.05. While SC-GRS has the lowest type I error, EV- is still preferable than OR-GRS (Supplemental Table S6).

Table 2 summarizes the results of significance tests of the pairwise contrasts of the different methods. For each table cell, if one method significantly outperforms the other, that method is listed. As these results show the weighted methods perform better than the SC-GRS in terms of power and C-statistic, but there is no significant difference between OR- and EV-GRS for these measures of performance.

**Table 2:** Significant winner^*^ in pair-wise method comparisons in terms of power, type I error, C-statistic and AIC.

**Pair-wise comparison**	**Power**	**Type I error**	**C-statistic**	**AIC**
SC-OR	OR		OR	
SC-EV	EV		EV	
OR-EV				

* *Significant winner denotes the method significantly outperforms with larger power and C-statistic, and smaller type I error and AIC (Tukey adjusted P-value <0.05). The blank means no significant difference is detected*.

## Discussion

The primary goal of our study was to explore how interaction and correlation could impact the risk prediction and to further determine the usefulness of genetic risk score as a predictive model that allows for gene-gene interaction, assuming sample sizes and genetic effect sizes likely to be encountered in real genetic studies. Since in real data the underlying disease mechanism is often unknown and is likely to vary across diseases, we try to employ relatively comprehensive simulation strategies to resemble potential complicated sources of interaction. Based on a wide range of simulation experiments, we observed that the weighted methods generally outperform the simple count method.

In simulation one, as expected, we observed a clear and intuitive relationship among effect size, MAF, heritability and power for all methods. For instance, in the case of MAF3 = MAF4 = 0.4 and ES3 = 0.2, *H*^2^_*G*3*G*4_ increases from 0.2 to 0.9% when ES4 increases from 0.2 to 0.5 (models 1–4). In the case of MAF3 = MAF4 = 0.05 and ES4 = 0.8, *H*^2^_*G*3*G*4_ increases from 0.4 to 0.7% when ES3 increases from 0.2 to 0.8 (models 10–7). The increase of heritability is more rapid for common variants. In respect to the MAF and heritability, in the case of ES3 = ES4 = 0.5 and MAF3 = 0.4, *H*^2^_*G*3*G*4_ increases from 0.9 to 1.5% when MAF4 increases from 0.05 to 0.4 (models 31–19). In the case of ES3 = 0.2, ES4 = 0.8 and MAF4 = 0.05, *H*^2^_*G*3*G*4_ increases from 0.4 to 0.5% when MAF3 increases from 0.05 to 0.4 (models 10–16). It is consistent with the assumption that both effect size and MAF are essential to drive the heritability and the impact of effect size seems more obvious. We also observed a clear pattern of heritability and power. The power of SC-GRS has improved from 26 to 75 and that of weighted GRS from 26 to 85, as the total heritability increases from 0.5 to 1.7% (models 1–4). Therefore, it becomes easy to identify the risk model when the heritability is high.

**Figure 5:**
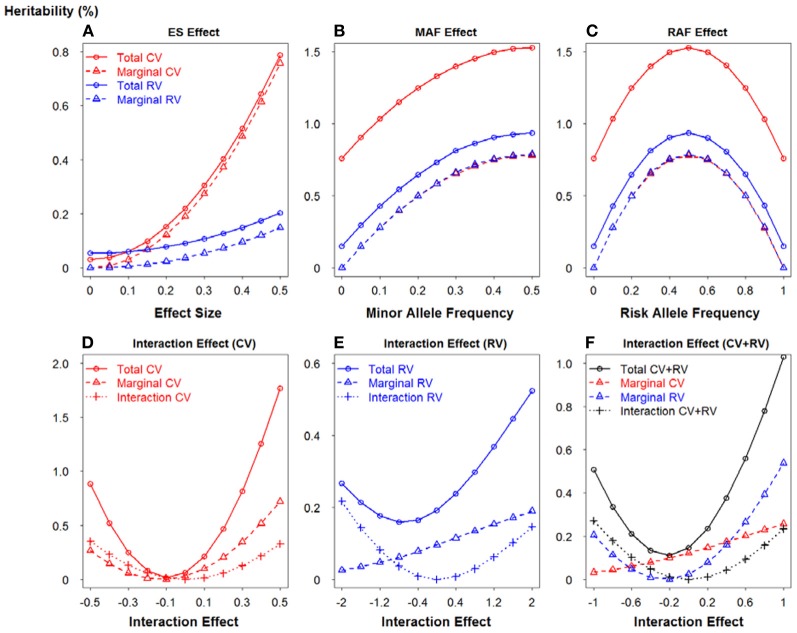
**Relationship between effect size, minor allele frequency, risk allele frequency, interaction effect and heritability**. **(A)** Relationship between heritability and effect size. **(B)** Relationship between heritability and minor allele frequency. **(C)** Relationship between heritability and risk allele frequency. **(D)** Relationship between heritability and interaction effect when SNPs are common variants. **(E)** Relationship between heritability and interaction effect when SNPs are rare variants. **(F)** Relationship between heritability and interaction effect when SNPs are common and rare variants.

To further demonstrate the relationship between the potential important factors and heritability, we utilized two-locus model to calculate the heritability due to total, marginal and interaction, according to formulas 13–15 respectively. Figure 5 portrays a clear relationship between effect size, minor allele frequency, risk allele frequency, interaction effect and heritability. Firstly, we assumed there is no interaction between two loci (panels **A–C**). Similarly, common variants have MAF = 0.4 and rare variants have MAF = 0.05. In panel **(A)**, ES1 was fixed at 0.1 if two loci were common, whereas ES1 was 0.3 if two loci were rare. When ES2 increases from 0 to 0.5, the total heritability and marginal heritability due to SNP 2 increase more rapidly for common than rare variants. In panels **(B–C)**, effect sizes for two loci were fixed at 0.5 and SNP 1 was considered as common and rare respectively. The total and marginal heritability due to SNP 2 increase as MAF2 increases from 0 to 0.5, and decline as risk allele frequency (RAF) 2 increases from 0.5 to 1. This relationship pattern further illustrates the validity of explained variance genetic risk score. Both the log scale of effect size and the square root of minor allele frequency are appropriate to explain the genetic variance due to SNP (that is heritability), and thus it is reasonable to incorporate both ES and MAF to construct the weight. Secondly, we investigated the relationship between interaction effect and heritability, and the results were shown in panels **(D–F)**. Both marginal effect sizes were set to 0.1 for panel **(D)** (common variants) and 0.4 for panel **(E)** (rare variants). In panel **(F)**, SNP 1 was common and SNP 2 was rare, but they were set as the same effect size 0.2. We considered both positive (β_12_ > 0) and negative interaction effect (β_12_ < 0). For positive interaction, when interaction effect increases, the total, marginal and interaction heritability increase consistently (panels **D–F**). Furthermore, as the interaction effect increases, the marginal heritability due to rare variant is more sensitive than that due to common variant (panel **F**). However, it is relatively complicated to account for negative interaction effect. The pattern depends on both marginal and interaction effect sizes. It also explains the complicated pattern for Figures 1, 2. As the negative interaction effect increases, the heritability may either increase or decrease, which in turn affects the power. Therefore, this relationship pattern provides some theoretical evidences for the weight construction method of EV-GRS, as well as the complicated pattern of the risk score model performance.

**Figure 6:**
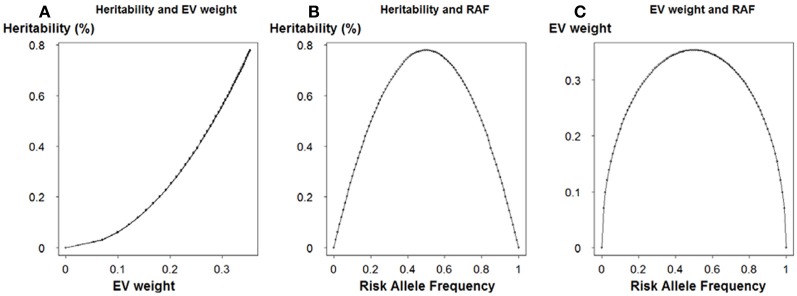
**Relationship between risk allele frequency, explained variance weight and heritability**. **(A)** Relationship between heritability and explained variance weight. **(B)** Relationship between heritability and risk allele frequency. **(C)** Relationship between explained variance weight and risk allele frequency.

To further address the role of allele frequency in the heritability, we applied one-locus model to calculate the heritability (formula 12). Effect size was fixed at 0.5 and risk allele frequency ranged from 0 to 1. Explained variance weight was obtained using formula 8, where the effect size was equivalent to log odds ratio in the logistic regression model. In Figure 6A, we observed a clear positive correlation between heritability and EV weight, which emphasized the role of allele frequency besides effect size. As to the relationship between heritability, EV weight and RAF, Figures 6B,C shared a similar pattern.

It should be noted that the performance of all three GRS models do not significantly differ for the models with rare variants with the same effect sizes, regardless of the strength or presence of interaction (Figure 1Bi). Referring of the total heritability of these models, it is understandable that the heritability due to interaction between SNPs 3 and 4 is very low, ranging from 0, 0.1 to 0.2%, while the total heritability is around 1.5% (models 7–9). This demonstrates the concept that the heritability due to only rare variants is limited, and thus GRS modeling may not work effectively in prediction with all the rare variants. Furthermore, in the scenario of all rare variants with similar effect sizes, weighted and unweighted methods are identical even if the interaction exists. Nevertheless, weighted methods are still preferable if different effect sizes exist, in all cases of MAF.

Understanding that current simulations may include a limited number of SNPs and low level interaction, as a follow up we also considered more complicated scenarios with six disease-causing SNPs. As shown in Supplemental Table S2, model 37 involved two two-way interactions, by varying the interaction term of SNPs 3 and 4 (β_34_) and that of SNPs 5 and 6 (β_56_). Similarly, model 38 involved three-way interactions, where SNPs 3, 4, and 5 were interacted to cause disease by specifying β_345_. The power improvement of the weighted GRS approaches were exaggerated compared with the SC-GRS. The details of results were presented in Supplemental Table S3. It seems that the impact of simulated effects may be amplified by the number of SNPs and the degree of interaction. Based on our empirical evidences, we expect these results could be generalized to larger models, but the extrapolations should be interpreted cautiously. Future studies should further evaluate the performance of these GRS methods for larger, and increasingly complex models.

In general, weighted and unweighted approaches are equivalent only if the true model is fully discovered, all causative SNPs have identical effect sizes and no interaction exists. However, it is almost impossible to satisfy all three requirements in practice. As the strength of interaction increases, the power of SC-GRS declines rapidly, while the advantage of weighted approaches becomes more obvious. If there are “noise” SNPs involving in the risk score, the weighted methods could limit the negative influence of the non-causative SNP. The performance of weighted method does not diminish when non-causative SNPs are added, and whether it is in strong or weak LD with true SNP. It is intuitive the performance of the weighted methods are greatly improved if the effect sizes of causative SNPs vary. As to the comparison of the weighted GRS approaches, EV- is preferred than OR-GRS with stronger power and lower type I error over these scenarios, but the difference is not statistically significant. Although it is believed that MAF is an important factor to explain the genetic variance and heritability, the effect size may still dominate the overall direction of the weight (Park et al., 2010). More comprehensive studies of MAF in the presence of interaction may be necessary.

In summary, our findings allow us to draw a consistent conclusion that weighted genetic risk score models are superior to the unweighted one overall and EV-GRS is the most robust approach, in the presence of potential genetic interactions, LD and false positive predictors. It provides some useful guidance for researchers in selecting an appropriate genetic risk score and advocates a wide implementation of the robust EV-GRS in real data analyses. It should be noted that beyond the discovery and identification of novel genetic variants, we are more focused on the follow-up direction to utilize these identified variants to predict the disease risk or the effectiveness and toxicity of interventions in pharmacogenetics study. Although the application of risk prediction in the complex disease has been limited due to complexity, several interesting evidences support application of prediction model in pharmacogenetic study is appealing and encouraging (Hess et al., 2006; Klein et al., 2009; Ritchie, 2012). Also, we are interested in the sensitiveness and robustness of the risk model. We should be cautious to use over-optimistic estimates for the risk factors, particularly in pharmacogenomics studies (Ritchie, 2012). In this sense, weighted methods perform robustly overall. Notably, it is reasonable and straightforward to assign more weights to important factors and limit the weights for seemingly noisy predictors.

Despite these solid conclusions, we recognized our study does not explicitly simulate more complex architectures and thus some inherent limitations remain to address. We only considered simple disease models involving marginal effects and two-locus interactions. Also, a limited number of scenarios of LD were examined. In future studies, it would be of great interest if we expand simulations to high-order gene-gene interactions, or even involving gene-environment interactions. Although it was not a thorough list of all complicated scenarios, our findings still provide insight that weighted risk model may play an vital role in the risk prediction when interaction or LD exists. Furthermore, current GRS models assume an additive model and independence of SNPs, which would rarely be the case. Despite the advantages of weighted GRS under the violation of these assumptions, the innovation of GRS that incorporates interaction term(s) will definitely be an important future direction. It is believed that to account for interaction or more complex architectures appropriately, the refinement and extension of risk models becomes an important priority in the human genetics.

### Conflict of interest statement

The authors declare that the research was conducted in the absence of any commercial or financial relationships that could be construed as a potential conflict of interest.
