# Supplementary material for: Evaluation of genetic risk score models in the presence of interaction and linkage disequilibrium
Source: Front Genet. 2013 Jul 23;4:138. doi: 10.3389/fgene.2013.00138 (PMC3719135; doi:10.3389/fgene.2013.00138)
Supplement: Supplementary file 1 [file 43602_Motsinger-Reif_DataSheet1.DOCX]

**Supplemental Files**

**Supplemental Table S1: Interaction model specifications with four or two deleterious SNPs.**

|  |  |  | MAF | | | | Effect Size | | | | | Heritability (%) | | | | | | | |
| --- | --- | --- | --- | --- | --- | --- | --- | --- | --- | --- | --- | --- | --- | --- | --- | --- | --- | --- | --- |
| MAF | ES | Model | f1 | f2 | f3 | f4 | b1 | b2 | b3 | b4 | b34 | Total | G1 | G2 | G3 | G4 | G1G2 | G3G4 | Int_G3G4 |
| CV | Same | 1 | 0.4 | 0.4 | 0.4 | 0.4 | 0.2 | 0.2 | 0.2 | 0.2 | 0 | 0.49 | 0.12 | 0.12 | 0.12 | 0.12 | 0.24 | 0.24 | 0.00 |
|  |  | 2 | 0.4 | 0.4 | 0.4 | 0.4 | 0.2 | 0.2 | 0.2 | 0.2 | -0.8 | 2.20 | 0.12 | 0.12 | 0.55 | 0.55 | 0.24 | 1.97 | 0.86 |
|  |  | 3 | 0.4 | 0.4 | 0.4 | 0.4 | 0.2 | 0.2 | 0.2 | 0.2 | -1.2 | 4.86 | 0.11 | 0.11 | 1.47 | 1.47 | 0.22 | 4.63 | 1.68 |
|  | Diff | 4 | 0.4 | 0.4 | 0.4 | 0.4 | 0.2 | 0.5 | 0.2 | 0.5 | 0 | 1.73 | 0.12 | 0.75 | 0.12 | 0.75 | 0.86 | 0.86 | 0.00 |
|  |  | 5 | 0.4 | 0.4 | 0.4 | 0.4 | 0.2 | 0.5 | 0.2 | 0.5 | -0.8 | 2.35 | 0.12 | 0.74 | 0.56 | 0.06 | 0.86 | 1.49 | 0.88 |
|  |  | 6 | 0.4 | 0.4 | 0.4 | 0.4 | 0.2 | 0.5 | 0.2 | 0.5 | -1.2 | 4.69 | 0.11 | 0.71 | 1.54 | 0.54 | 0.82 | 3.87 | 1.78 |
| RV | Same | 7 | 0.05 | 0.05 | 0.05 | 0.05 | 0.8 | 0.8 | 0.8 | 0.8 | 0 | 1.45 | 0.36 | 0.36 | 0.36 | 0.36 | 0.73 | 0.73 | 0.00 |
|  |  | 8 | 0.05 | 0.05 | 0.05 | 0.05 | 0.8 | 0.8 | 0.8 | 0.8 | -1 | 1.35 | 0.36 | 0.36 | 0.28 | 0.28 | 0.73 | 0.62 | 0.05 |
|  |  | 9 | 0.05 | 0.05 | 0.05 | 0.05 | 0.8 | 0.8 | 0.8 | 0.8 | -2 | 1.36 | 0.36 | 0.36 | 0.21 | 0.21 | 0.73 | 0.63 | 0.22 |
|  | Diff | 10 | 0.05 | 0.05 | 0.05 | 0.05 | 0.2 | 0.8 | 0.2 | 0.8 | 0 | 0.79 | 0.02 | 0.37 | 0.02 | 0.37 | 0.39 | 0.39 | 0.00 |
|  |  | 11 | 0.05 | 0.05 | 0.05 | 0.05 | 0.2 | 0.8 | 0.2 | 0.8 | -1 | 0.74 | 0.02 | 0.37 | 0.01 | 0.28 | 0.40 | 0.35 | 0.06 |
|  |  | 12 | 0.05 | 0.05 | 0.05 | 0.05 | 0.2 | 0.8 | 0.2 | 0.8 | -2 | 0.82 | 0.02 | 0.37 | 0.00 | 0.21 | 0.39 | 0.43 | 0.22 |
| CV+RV | Same | 13 | 0.4 | 0.05 | 0.4 | 0.05 | 0.5 | 0.5 | 0.5 | 0.5 | 0 | 1.78 | 0.75 | 0.15 | 0.75 | 0.15 | 0.89 | 0.89 | 0.00 |
|  |  | 14 | 0.4 | 0.05 | 0.4 | 0.05 | 0.5 | 0.5 | 0.5 | 0.5 | -1 | 1.70 | 0.75 | 0.15 | 0.48 | 0.05 | 0.89 | 0.81 | 0.28 |
|  |  | 15 | 0.4 | 0.05 | 0.4 | 0.05 | 0.5 | 0.5 | 0.5 | 0.5 | -3 | 3.96 | 0.72 | 0.14 | 0.22 | 1.34 | 0.86 | 3.10 | 1.54 |
|  | Diff | 16 | 0.4 | 0.05 | 0.4 | 0.05 | 0.2 | 0.8 | 0.2 | 0.8 | 0 | 0.99 | 0.12 | 0.37 | 0.12 | 0.37 | 0.49 | 0.49 | 0.00 |
|  |  | 17 | 0.4 | 0.05 | 0.4 | 0.05 | 0.2 | 0.8 | 0.2 | 0.8 | -1 | 0.81 | 0.12 | 0.38 | 0.03 | 0.00 | 0.50 | 0.31 | 0.28 |
|  |  | 18 | 0.4 | 0.05 | 0.4 | 0.05 | 0.2 | 0.8 | 0.2 | 0.8 | -3 | 3.05 | 0.12 | 0.36 | 0.01 | 0.95 | 0.48 | 2.57 | 1.61 |

**Supplemental Table S1 (continued)**

|  |  |  | MAF | | | | Effect Size | | | | | Heritability (%) | | | | | | | |
| --- | --- | --- | --- | --- | --- | --- | --- | --- | --- | --- | --- | --- | --- | --- | --- | --- | --- | --- | --- |
| MAF | ES | Model | f1 | f2 | f3 | f4 | b1 | b2 | b3 | b4 | b34 | Total | G1 | G2 | G3 | G4 | G1G2 | G3G4 | Int_G3G4 |
| CV | Same | 19 | 0.4 | 0.4 | 0.4 | 0.4 | 0 | 0 | 0.5 | 0.5 | 0 | 1.50 | 0.00 | 0.00 | 0.75 | 0.75 | 0.00 | 1.50 | 0.00 |
|  |  | 20 | 0.4 | 0.4 | 0.4 | 0.4 | 0 | 0 | 0.5 | 0.5 | -0.8 | 1.03 | 0.00 | 0.00 | 0.06 | 0.06 | 0.00 | 1.03 | 0.91 |
|  |  | 21 | 0.4 | 0.4 | 0.4 | 0.4 | 0 | 0 | 0.5 | 0.5 | -1.2 | 3.07 | 0.00 | 0.00 | 0.58 | 0.58 | 0.00 | 3.07 | 1.90 |
|  | Diff | 22 | 0.4 | 0.4 | 0.4 | 0.4 | 0 | 0 | 0.2 | 0.5 | 0 | 0.88 | 0.00 | 0.00 | 0.12 | 0.76 | 0.00 | 0.88 | 0.00 |
|  |  | 23 | 0.4 | 0.4 | 0.4 | 0.4 | 0 | 0 | 0.2 | 0.5 | -0.8 | 1.52 | 0.00 | 0.00 | 0.57 | 0.06 | 0.00 | 1.52 | 0.89 |
|  |  | 24 | 0.4 | 0.4 | 0.4 | 0.4 | 0 | 0 | 0.2 | 0.5 | -1.2 | 3.89 | 0.00 | 0.00 | 1.55 | 0.54 | 0.00 | 3.89 | 1.80 |
| RV | Same | 25 | 0.05 | 0.05 | 0.05 | 0.05 | 0 | 0 | 1 | 1 | 0 | 1.14 | 0.00 | 0.00 | 0.57 | 0.57 | 0.00 | 1.14 | 0.00 |
|  |  | 26 | 0.05 | 0.05 | 0.05 | 0.05 | 0 | 0 | 1 | 1 | -1 | 1.00 | 0.00 | 0.00 | 0.47 | 0.47 | 0.00 | 1.00 | 0.05 |
|  |  | 27 | 0.05 | 0.05 | 0.05 | 0.05 | 0 | 0 | 1 | 1 | -2 | 0.96 | 0.00 | 0.00 | 0.37 | 0.37 | 0.00 | 0.96 | 0.22 |
|  | Diff | 28 | 0.05 | 0.05 | 0.05 | 0.05 | 0 | 0 | 0.2 | 0.8 | 0 | 0.40 | 0.00 | 0.00 | 0.02 | 0.38 | 0.00 | 0.40 | 0.00 |
|  |  | 29 | 0.05 | 0.05 | 0.05 | 0.05 | 0 | 0 | 0.2 | 0.8 | -1 | 0.35 | 0.00 | 0.00 | 0.01 | 0.29 | 0.00 | 0.35 | 0.06 |
|  |  | 30 | 0.05 | 0.05 | 0.05 | 0.05 | 0 | 0 | 0.2 | 0.8 | -2 | 0.43 | 0.00 | 0.00 | 0.00 | 0.21 | 0.00 | 0.43 | 0.22 |
| CV+RV | Same | 31 | 0.4 | 0.05 | 0.4 | 0.05 | 0 | 0 | 0.5 | 0.5 | 0 | 0.90 | 0.00 | 0.00 | 0.76 | 0.15 | 0.00 | 0.90 | 0.00 |
|  |  | 32 | 0.4 | 0.05 | 0.4 | 0.05 | 0 | 0 | 0.5 | 0.5 | -1 | 0.82 | 0.00 | 0.00 | 0.49 | 0.05 | 0.00 | 0.82 | 0.28 |
|  |  | 33 | 0.4 | 0.05 | 0.4 | 0.05 | 0 | 0 | 0.5 | 0.5 | -3 | 3.16 | 0.00 | 0.00 | 0.22 | 1.37 | 0.00 | 3.16 | 1.57 |
|  | Diff | 34 | 0.4 | 0.05 | 0.4 | 0.05 | 0 | 0 | 0.3 | 1 | 0 | 0.85 | 0.00 | 0.00 | 0.27 | 0.58 | 0.00 | 0.85 | 0.00 |
|  |  | 35 | 0.4 | 0.05 | 0.4 | 0.05 | 0 | 0 | 0.3 | 1 | -1 | 0.43 | 0.00 | 0.00 | 0.12 | 0.02 | 0.00 | 0.43 | 0.28 |
|  |  | 36 | 0.4 | 0.05 | 0.4 | 0.05 | 0 | 0 | 0.3 | 1 | -3 | 2.47 | 0.00 | 0.00 | 0.01 | 0.74 | 0.00 | 2.47 | 1.72 |

**Supplemental Table S2: Interaction model specifications with six deleterious SNPs.**

|  | MAF | | | | | | Effect Size | | | | | | | | | Heritability (%) | | | | | | | | | |
| --- | --- | --- | --- | --- | --- | --- | --- | --- | --- | --- | --- | --- | --- | --- | --- | --- | --- | --- | --- | --- | --- | --- | --- | --- | --- |
| Model | f1 | f2 | f3 | f4 | f5 | f6 | b1 | b2 | b3 | b4 | b5 | b6 | b34 | b56 | b345 | Total | G1 | G2 | G3 | G4 | G5 | G6 | Int_G3G4 | Int_G5G6 | Int_G3G4G5 |
| 37 | 0.4 | 0.05 | 0.4 | 0.05 | 0.4 | 0.05 | 0.2 | 0.2 | 0.2 | 0.2 | 0.2 | 0.2 | -1 | -2 | 0 | 2.40 | 0.12 | 0.02 | 0.03 | 0.20 | 0.00 | 0.92 | 0.26 | 0.84 | 0.00 |
| 38 | 0.4 | 0.05 | 0.4 | 0.05 | 0.4 | 0.05 | 0.2 | 0.2 | 0.2 | 0.2 | 0.2 | 0.2 | 0 | 0 | -4 | 3.21 | 0.12 | 0.02 | 0.02 | 0.99 | 0.02 | 0.02 | 0.00 | 0.00 | 2.02 |

**Supplemental Table S3: Interaction model results.**

|  |  |  | Power | | | Type I Error | | | C | | | AIC | | |
| --- | --- | --- | --- | --- | --- | --- | --- | --- | --- | --- | --- | --- | --- | --- |
| MAF | ES | Model | SC | OR | EV | SC | OR | EV | SC | OR | EV | SC | OR | EV |
| CV | Same | 1 | 26 | 26 | 26 | 4 | 6 | 6 | 0.538 | 0.538 | 0.538 | 694.140 | 694.136 | 694.136 |
|  |  | 2 | 11 | 71 | 71 | 4 | 5 | 5 | 0.526 | 0.561 | 0.561 | 695.491 | 689.895 | 689.895 |
|  |  | 3 | 49 | 99 | 99 | 5 | 9 | 9 | 0.548 | 0.599 | 0.599 | 692.160 | 680.143 | 680.143 |
|  | Diff | 4 | 75 | 85 | 85 | 6 | 7 | 7 | 0.564 | 0.571 | 0.571 | 689.667 | 688.295 | 688.295 |
|  |  | 5 | 7 | 76 | 76 | 2 | 3 | 3 | 0.520 | 0.568 | 0.568 | 696.132 | 689.013 | 689.013 |
|  |  | 6 | 15 | 99 | 99 | 7 | 2 | 2 | 0.524 | 0.594 | 0.594 | 695.571 | 681.744 | 681.744 |
| RV | Same | 7 | 77 | 78 | 78 | 5 | 6 | 6 | 0.558 | 0.558 | 0.558 | 688.139 | 688.146 | 688.145 |
|  |  | 8 | 72 | 72 | 72 | 5 | 3 | 3 | 0.553 | 0.554 | 0.554 | 689.238 | 689.091 | 689.091 |
|  |  | 9 | 64 | 65 | 66 | 4 | 7 | 7 | 0.552 | 0.552 | 0.552 | 690.606 | 690.420 | 690.419 |
|  | Diff | 10 | 45 | 56 | 56 | 5 | 5 | 5 | 0.537 | 0.540 | 0.540 | 693.043 | 691.937 | 691.938 |
|  |  | 11 | 31 | 48 | 48 | 7 | 7 | 7 | 0.532 | 0.535 | 0.535 | 694.155 | 692.841 | 692.841 |
|  |  | 12 | 28 | 35 | 34 | 7 | 9 | 9 | 0.532 | 0.532 | 0.532 | 694.322 | 693.177 | 693.178 |
| CV+RV | Same | 13 | 81 | 81 | 81 | 8 | 10 | 8 | 0.573 | 0.574 | 0.573 | 686.850 | 686.845 | 687.348 |
|  |  | 14 | 68 | 76 | 74 | 6 | 6 | 5 | 0.557 | 0.569 | 0.567 | 690.891 | 688.804 | 689.071 |
|  |  | 15 | 34 | 94 | 94 | 7 | 6 | 7 | 0.542 | 0.593 | 0.585 | 693.883 | 683.563 | 685.288 |
|  | Diff | 16 | 42 | 55 | 49 | 5 | 7 | 8 | 0.542 | 0.551 | 0.549 | 693.232 | 691.243 | 691.970 |
|  |  | 17 | 24 | 29 | 33 | 7 | 7 | 7 | 0.533 | 0.536 | 0.536 | 694.618 | 693.552 | 693.901 |
|  |  | 18 | 4 | 75 | 73 | 3 | 6 | 4 | 0.521 | 0.556 | 0.557 | 696.126 | 688.857 | 689.412 |

**Supplemental Table S3 (continued)**

|  |  |  | Power | | | Type I Error | | | C | | | AIC | | |
| --- | --- | --- | --- | --- | --- | --- | --- | --- | --- | --- | --- | --- | --- | --- |
| MAF | ES | Model | SC | OR | EV | SC | OR | EV | SC | OR | EV | SC | OR | EV |
| CV | Same | 19 | 54 | 84 | 84 | 4 | 8 | 8 | 0.551 | 0.571 | 0.571 | 692.052 | 688.131 | 688.131 |
|  |  | 20 | 9 | 14 | 14 | 4 | 3 | 3 | 0.521 | 0.523 | 0.523 | 695.952 | 695.564 | 695.564 |
|  |  | 21 | 47 | 72 | 72 | 2 | 3 | 3 | 0.545 | 0.558 | 0.558 | 693.024 | 689.856 | 689.855 |
|  | Diff | 22 | 24 | 54 | 54 | 5 | 7 | 7 | 0.537 | 0.553 | 0.553 | 694.355 | 691.869 | 691.869 |
|  |  | 23 | 19 | 41 | 41 | 8 | 9 | 9 | 0.529 | 0.543 | 0.543 | 694.980 | 693.412 | 693.412 |
|  |  | 24 | 67 | 97 | 97 | 4 | 8 | 8 | 0.557 | 0.579 | 0.579 | 690.782 | 685.717 | 685.717 |
| RV | Same | 25 | 39 | 69 | 69 | 6 | 5 | 5 | 0.538 | 0.544 | 0.544 | 692.812 | 689.894 | 689.894 |
|  |  | 26 | 36 | 61 | 61 | 5 | 10 | 10 | 0.535 | 0.541 | 0.541 | 693.523 | 690.981 | 690.981 |
|  |  | 27 | 31 | 58 | 58 | 3 | 6 | 7 | 0.534 | 0.539 | 0.539 | 694.121 | 691.722 | 691.723 |
|  | Diff | 28 | 12 | 28 | 28 | 4 | 6 | 7 | 0.518 | 0.526 | 0.526 | 695.629 | 694.124 | 694.124 |
|  |  | 29 | 10 | 22 | 22 | 3 | 5 | 5 | 0.518 | 0.523 | 0.523 | 695.924 | 694.759 | 694.759 |
|  |  | 30 | 10 | 19 | 19 | 4 | 4 | 4 | 0.520 | 0.520 | 0.520 | 695.850 | 695.126 | 695.125 |
| CV+RV | Same | 31 | 35 | 51 | 50 | 3 | 6 | 4 | 0.540 | 0.551 | 0.551 | 693.722 | 691.688 | 691.871 |
|  |  | 32 | 19 | 39 | 44 | 5 | 7 | 7 | 0.527 | 0.544 | 0.541 | 695.337 | 693.104 | 693.245 |
|  |  | 33 | 7 | 79 | 81 | 5 | 4 | 7 | 0.520 | 0.571 | 0.571 | 695.966 | 686.566 | 687.477 |
|  | Diff | 34 | 25 | 54 | 49 | 5 | 4 | 3 | 0.533 | 0.548 | 0.547 | 694.556 | 692.229 | 692.860 |
|  |  | 35 | 5 | 14 | 18 | 6 | 6 | 4 | 0.526 | 0.527 | 0.528 | 695.814 | 695.252 | 695.290 |
|  |  | 36 | 7 | 56 | 58 | 2 | 6 | 9 | 0.521 | 0.547 | 0.547 | 695.956 | 691.772 | 691.827 |
| CV+RV | Same | 37 | 2 | 72 | 67 | 6 | 4 | 3 | 0.518 | 0.562 | 0.559 | 696.311 | 689.121 | 689.99 |
|  |  | 38 | 6 | 69 | 64 | 8 | 6 | 5 | 0.519 | 0.557 | 0.558 | 696.209 | 689.635 | 690.211 |

**Supplemental Table S4: Linkage disequilibrium model SNP 3 genotype frequencies specifications.**

|  | Dependence of |  |  | SNP 3 |  |
| --- | --- | --- | --- | --- | --- |
| Scenario | SNPs 2 and 3 | SNP 2 | CC | Cc | cc |
| 1 | Dependent | BB | 0.8 | 0.1 | 0.1 |
|  |  | Bb | 0.1 | 0.8 | 0.1 |
|  |  | bb | 0.1 | 0.1 | 0.8 |
|  |  |  |  |  |  |
| 2 | Independent | BB/Bb/bb | 0.25 | 0.5 | 0.25 |

**Supplemental Table S5: Linkage disequilibrium model specifications and results when SNPs 2 and 3 are dependent or independent.**

|  |  |  |  | Power | | | Type I Error | | | C | | | AIC | | |  |  |  |  |  |
| --- | --- | --- | --- | --- | --- | --- | --- | --- | --- | --- | --- | --- | --- | --- | --- | --- | --- | --- | --- | --- |
| MAF | ES | Dep | Model | SC | OR | EV | SC | OR | EV | SC | OR | EV | SC | OR | EV | MAF1 | MAF2 | RR1 | RR2 | k |
| CV | Same | True | 1 | 53 | 53 | 53 | 6 | 6 | 8 | 0.557 | 0.557 | 0.557 | 553.314 | 553.319 | 553.319 | 0.4 | 0.4 | 1.5 | 1.5 | 0.1 |
|  |  | True +Dep | 2 | 41 | 53 | 53 | 5 | 6 | 7 | 0.553 | 0.557 | 0.557 | 553.969 | 553.333 | 553.334 | 0.4 | 0.4 | 1.5 | 1.5 | 0.1 |
|  | Diff | True | 3 | 51 | 64 | 64 | 8 | 6 | 6 | 0.556 | 0.565 | 0.565 | 553.353 | 552.236 | 552.236 | 0.4 | 0.4 | 1.25 | 1.75 | 0.1 |
|  |  | True +Dep | 4 | 54 | 63 | 63 | 5 | 5 | 6 | 0.559 | 0.565 | 0.565 | 553.209 | 552.257 | 552.257 | 0.4 | 0.4 | 1.25 | 1.75 | 0.1 |
| RV | Same | True | 5 | 60 | 61 | 61 | 8 | 8 | 8 | 0.544 | 0.544 | 0.544 | 553.116 | 553.117 | 553.117 | 0.05 | 0.05 | 2 | 2 | 0.1 |
|  |  | True +Dep | 6 | 33 | 60 | 61 | 5 | 8 | 8 | 0.537 | 0.544 | 0.545 | 555.475 | 553.112 | 553.110 | 0.05 | 0.05 | 2 | 2 | 0.1 |
|  | Diff | True | 7 | 21 | 24 | 25 | 9 | 6 | 6 | 0.526 | 0.525 | 0.525 | 556.224 | 555.902 | 555.897 | 0.05 | 0.05 | 1.25 | 1.75 | 0.1 |
|  |  | True +Dep | 8 | 8 | 23 | 23 | 5 | 6 | 7 | 0.526 | 0.526 | 0.528 | 556.700 | 555.903 | 555.909 | 0.05 | 0.05 | 1.25 | 1.75 | 0.1 |
| CV+RV | Same | True | 9 | 48 | 48 | 48 | 8 | 8 | 5 | 0.550 | 0.550 | 0.550 | 554.230 | 554.230 | 554.345 | 0.4 | 0.05 | 1.5 | 1.5 | 0.1 |
|  |  | True +Dep | 10 | 34 | 46 | 47 | 7 | 8 | 8 | 0.544 | 0.549 | 0.549 | 555.366 | 554.272 | 554.388 | 0.4 | 0.05 | 1.5 | 1.5 | 0.1 |
|  | Diff | True | 11 | 22 | 21 | 22 | 9 | 10 | 9 | 0.535 | 0.537 | 0.537 | 556.029 | 555.931 | 555.976 | 0.4 | 0.05 | 1.25 | 1.75 | 0.1 |
|  |  | True +Dep | 12 | 16 | 20 | 22 | 9 | 9 | 9 | 0.533 | 0.537 | 0.538 | 556.491 | 555.926 | 555.982 | 0.4 | 0.05 | 1.25 | 1.75 | 0.1 |
| RV+CV | Same | True | 13 | 46 | 43 | 46 | 5 | 4 | 4 | 0.548 | 0.547 | 0.548 | 554.591 | 554.656 | 554.600 | 0.05 | 0.4 | 1.5 | 1.5 | 0.1 |
|  |  | True +Dep | 14 | 34 | 43 | 46 | 7 | 5 | 4 | 0.543 | 0.548 | 0.548 | 555.217 | 554.656 | 554.595 | 0.05 | 0.4 | 1.5 | 1.5 | 0.1 |
|  | Diff | True | 15 | 24 | 24 | 27 | 7 | 5 | 6 | 0.534 | 0.536 | 0.535 | 556.201 | 555.814 | 556.061 | 0.05 | 0.4 | 1.75 | 1.25 | 0.1 |
|  |  | True +Dep | 16 | 12 | 24 | 27 | 5 | 5 | 6 | 0.531 | 0.536 | 0.536 | 556.671 | 555.811 | 556.058 | 0.05 | 0.4 | 1.75 | 1.25 | 0.1 |

**Supplemental Table S5 (continued)**

|  |  |  |  | Power | | | Type I Error | | | C | | | AIC | | |  |  |  |  |  |
| --- | --- | --- | --- | --- | --- | --- | --- | --- | --- | --- | --- | --- | --- | --- | --- | --- | --- | --- | --- | --- |
| MAF | ES | Dep | Model | SC | OR | EV | SC | OR | EV | SC | OR | EV | SC | OR | EV | MAF1 | MAF2 | RR1 | RR2 | k |
| CV | Same | True | 17 | 52 | 52 | 52 | 2 | 3 | 3 | 0.554 | 0.554 | 0.554 | 553.855 | 553.847 | 553.847 | 0.4 | 0.4 | 1.5 | 1.5 | 0.1 |
|  |  | True+Indep | 18 | 40 | 52 | 52 | 4 | 3 | 3 | 0.545 | 0.554 | 0.554 | 555.125 | 553.847 | 553.847 | 0.4 | 0.4 | 1.5 | 1.5 | 0.1 |
|  | Diff | True | 19 | 56 | 66 | 66 | 5 | 4 | 4 | 0.554 | 0.562 | 0.562 | 553.885 | 553.004 | 553.003 | 0.4 | 0.4 | 1.25 | 1.75 | 0.1 |
|  |  | True+Indep | 20 | 38 | 66 | 66 | 3 | 4 | 4 | 0.548 | 0.562 | 0.562 | 554.933 | 553.002 | 553.001 | 0.4 | 0.4 | 1.25 | 1.75 | 0.1 |
| RV | Same | True | 21 | 59 | 59 | 59 | 5 | 5 | 5 | 0.544 | 0.544 | 0.543 | 552.878 | 552.879 | 552.877 | 0.05 | 0.05 | 2 | 2 | 0.1 |
|  |  | True+Indep | 22 | 17 | 59 | 59 | 6 | 5 | 5 | 0.531 | 0.542 | 0.542 | 556.255 | 552.881 | 552.883 | 0.05 | 0.05 | 2 | 2 | 0.1 |
|  | Diff | True | 23 | 25 | 30 | 30 | 5 | 4 | 4 | 0.529 | 0.529 | 0.528 | 555.927 | 555.579 | 555.576 | 0.05 | 0.05 | 1.25 | 1.75 | 0.1 |
|  |  | True+Indep | 24 | 9 | 31 | 30 | 2 | 5 | 5 | 0.525 | 0.526 | 0.527 | 557.274 | 555.638 | 555.718 | 0.05 | 0.05 | 1.25 | 1.75 | 0.1 |
| CV+RV | Same | True | 25 | 36 | 38 | 39 | 3 | 3 | 1 | 0.545 | 0.543 | 0.545 | 555.253 | 555.325 | 555.166 | 0.4 | 0.05 | 1.5 | 1.5 | 0.1 |
|  |  | True+Indep | 26 | 20 | 36 | 40 | 4 | 3 | 1 | 0.535 | 0.543 | 0.545 | 556.339 | 555.366 | 555.214 | 0.4 | 0.05 | 1.5 | 1.5 | 0.1 |
|  | Diff | True | 27 | 33 | 29 | 32 | 5 | 6 | 5 | 0.538 | 0.539 | 0.539 | 555.548 | 555.371 | 555.488 | 0.4 | 0.05 | 1.25 | 1.75 | 0.1 |
|  |  | True+Indep | 28 | 24 | 29 | 31 | 4 | 6 | 6 | 0.534 | 0.539 | 0.539 | 556.314 | 555.375 | 555.486 | 0.4 | 0.05 | 1.25 | 1.75 | 0.1 |
| RV+CV | Same | True | 29 | 42 | 41 | 41 | 4 | 4 | 3 | 0.546 | 0.546 | 0.546 | 554.708 | 554.711 | 554.819 | 0.05 | 0.4 | 1.5 | 1.5 | 0.1 |
|  |  | True+Indep | 30 | 27 | 41 | 41 | 5 | 3 | 3 | 0.538 | 0.547 | 0.546 | 555.853 | 554.704 | 554.809 | 0.05 | 0.4 | 1.5 | 1.5 | 0.1 |
|  | Diff | True | 31 | 20 | 24 | 20 | 8 | 6 | 10 | 0.533 | 0.534 | 0.534 | 556.273 | 555.937 | 556.139 | 0.05 | 0.4 | 1.75 | 1.25 | 0.1 |
|  |  | True+Indep | 32 | 8 | 24 | 20 | 4 | 5 | 6 | 0.530 | 0.534 | 0.535 | 556.948 | 555.962 | 556.168 | 0.05 | 0.4 | 1.75 | 1.25 | 0.1 |

**Supplemental Table S6: Means of power, type I error, C and AIC across all scenarios.**

| Method | Power | Type I Error | C | AIC |
| --- | --- | --- | --- | --- |
| SC-GRS | 32.571 | 5.230 | 0.538 | 630.409 |
| OR-GRS | 51.714 | 5.743 | 0.549 | 628.230 |
| EV-GRS | 51.857 | 5.729 | 0.549 | 628.357 |
| *P*-value* | 4.1E-08 | 0.219 | 2.0E-05 | 0.978 |

*: *P*-value<0.05 indicates methods differ significantly, using ANOVA test.

**Supplemental Table S7: Pair-wise methods comparisons of power, type I error, C and AIC across all scenarios.**

| Pair-wise | Power | | Type I Error | | C | | AIC | |
| --- | --- | --- | --- | --- | --- | --- | --- | --- |
| Methods Comparison | Difference | *P*-value* | Difference | *P*-value* | Difference | *P*-value* | Difference | *P*-value* |
| SC - OR | -19.143 | 1.1E-06 | -0.514 | 0.288 | -0.011 | 1.3E-04 | 2.179 | 0.981 |
| SC - EV | -19.286 | 9.4E-07 | -0.500 | 0.313 | -0.011 | 1.7E-04 | 2.053 | 0.983 |
| OR - EV | -0.143 | 0.999 | 0.014 | 1.000 | 0.000 | 0.998 | -0.126 | 1.000 |
| SC - Cut-off (0.05) |  |  | 0.229 | 0.860 |  |  |  |  |
| OR - Cut-off (0.05) |  |  | 0.743 | 0.053 |  |  |  |  |
| EV - Cut-off (0.05) |  |  | 0.729 | 0.060 |  |  |  |  |

*: *P*-value<0.05 indicates methods differ significantly, using pair-wise comparison and adjusted by Tukey method.
